# Supplementary material for: A Positive Feedback DNA-PK/MYT1L-CXCR1-ERK1/2 Proliferative Signaling Loop in Glioblastoma
Source: Int J Mol Sci. 2025 May 6;26(9):4398. doi: 10.3390/ijms26094398 (PMC12072392; doi:10.3390/ijms26094398)
Supplement: Supplementary file 1 [file ijms-26-04398-s001.zip › 0716_2020 MYT1L_SUPPL.pptx]

## Slide 1
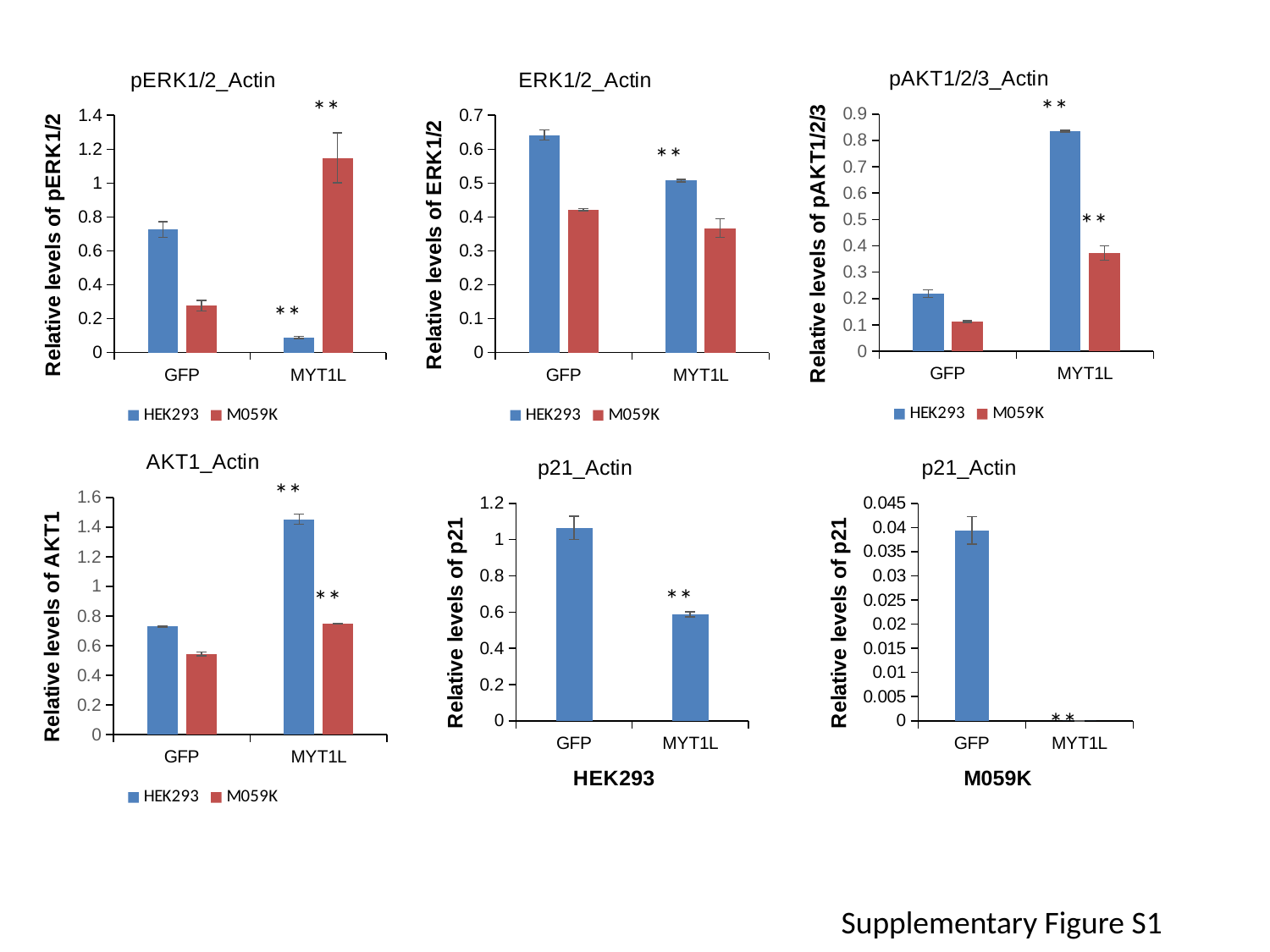

### Chart: pAKT1/2/3_Actin
| Category | HEK293 | M059K |
|---|---|---|
| GFP | 0.21857369171429383 | 0.1120453191676994 |
| MYT1L | 0.8350898016042754 | 0.372164686767525 |
### Chart: pERK1/2_Actin
| Category | HEK293 | M059K |
|---|---|---|
| GFP | 0.7262935567290217 | 0.2771874684999608 |
| MYT1L | 0.08949764458906993 | 1.1482085818976657 |
### Chart: ERK1/2_Actin
| Category | HEK293 | M059K |
|---|---|---|
| GFP | 0.6416245116463956 | 0.42164948197094415 |
| MYT1L | 0.5075057603783782 | 0.36677110212243375 |**
**
**
**
**
### Chart: AKT1_Actin
| Category | HEK293 | M059K |
|---|---|---|
| GFP | 0.7281940832505202 | 0.5439409372494509 |
| MYT1L | 1.4525155908264853 | 0.748737241875148 |
### Chart: p21_Actin
| Category | |
|---|---|
| GFP | 1.0650756542778879 |
| MYT1L | 0.5866414608598277 |
### Chart: p21_Actin
| Category | M059K |
|---|---|
| GFP | 0.039376569741802044 |
| MYT1L | 0.0 |**
**
**
**
Supplementary Figure S1

## Slide 2
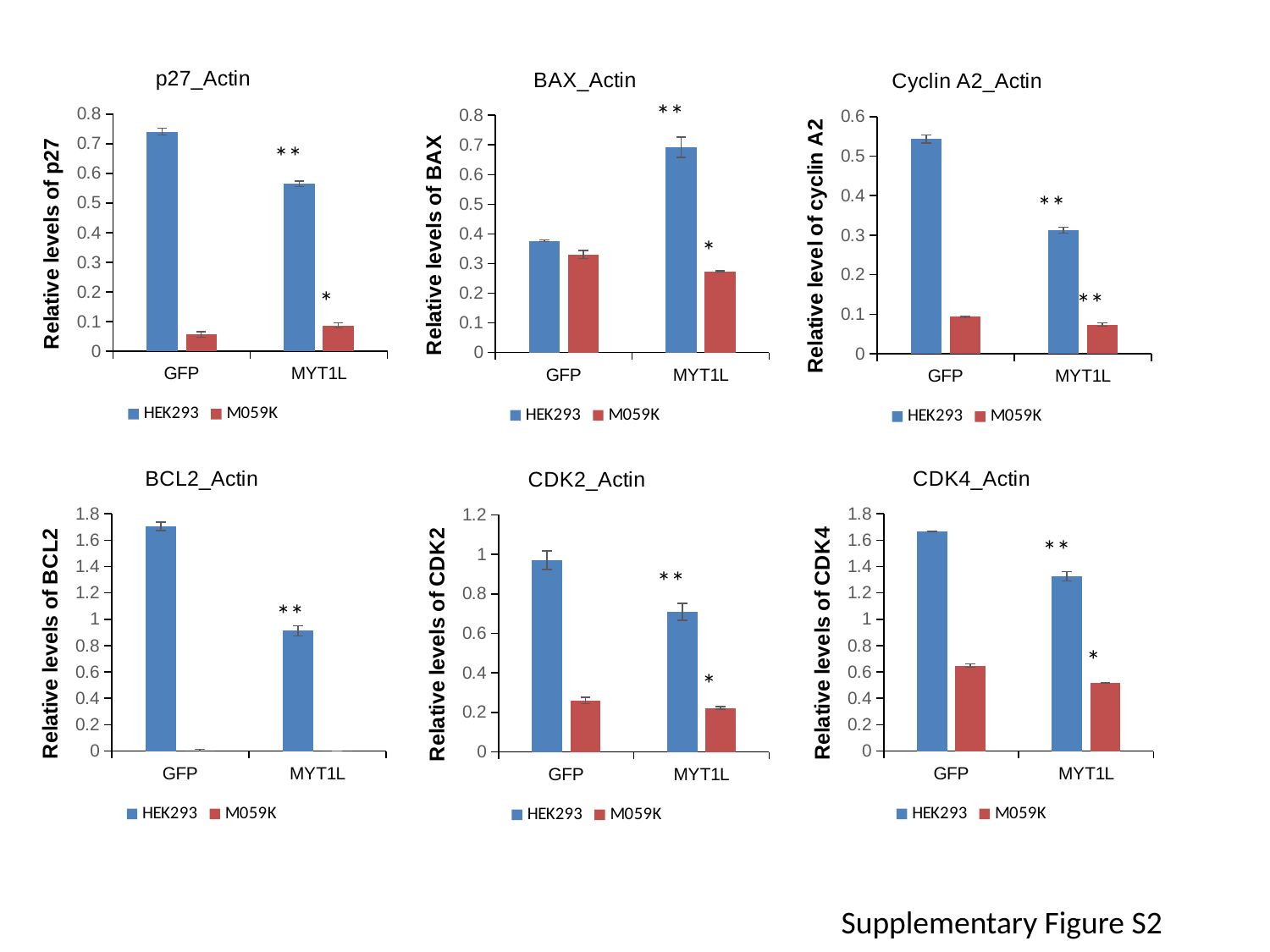

### Chart: p27_Actin
| Category | HEK293 | M059K |
|---|---|---|
| GFP | 0.7405487132009876 | 0.05678450259816308 |
| MYT1L | 0.5647459961387113 | 0.0869465066537455 |
### Chart: BAX_Actin
| Category | HEK293 | M059K |
|---|---|---|
| GFP | 0.3766254080154596 | 0.3301282654364625 |
| MYT1L | 0.6924868665266549 | 0.2734015607998084 |
### Chart: Cyclin A2_Actin
| Category | HEK293 | M059K |
|---|---|---|
| GFP | 0.5431240490414634 | 0.09351479336111834 |
| MYT1L | 0.3131043224445782 | 0.07396921778613107 |**
**
**
*
*
**
### Chart: BCL2_Actin
| Category | HEK293 | M059K |
|---|---|---|
| GFP | 1.7049298839408022 | 0.0 |
| MYT1L | 0.912608352718521 | 0.0 |
### Chart: CDK4_Actin
| Category | HEK293 | M059K |
|---|---|---|
| GFP | 1.6685795343835548 | 0.6480842567122034 |
| MYT1L | 1.3264268735471225 | 0.5178816454491934 |
### Chart: CDK2_Actin
| Category | HEK293 | M059K |
|---|---|---|
| GFP | 0.9692406550174187 | 0.261067630107441 |
| MYT1L | 0.7096344768904116 | 0.22266274171036882 |**
**
**
*
*
Supplementary Figure S2

## Slide 3
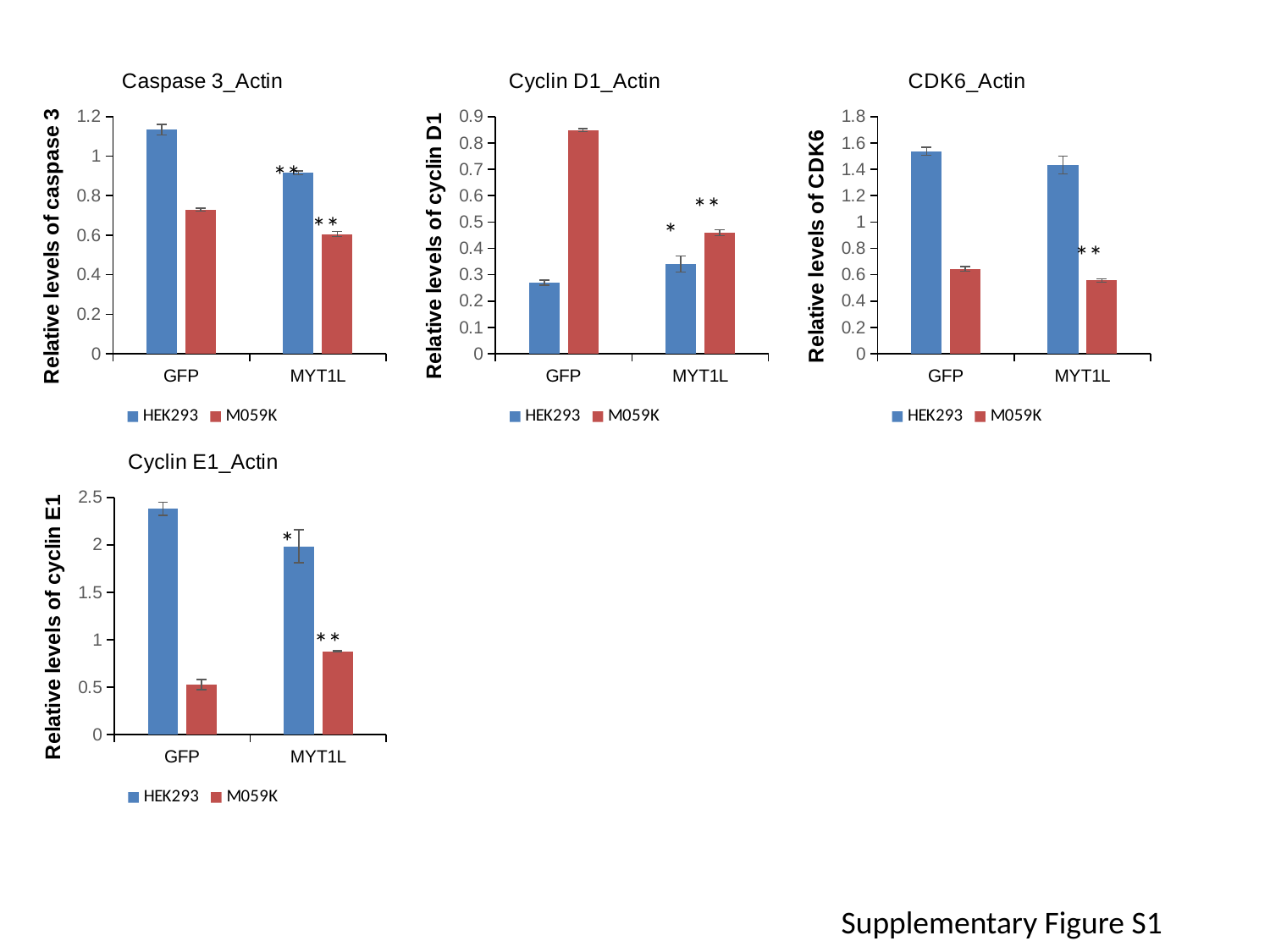

### Chart: Caspase 3_Actin
| Category | HEK293 | M059K |
|---|---|---|
| GFP | 1.1342091458859038 | 0.7287030997131801 |
| MYT1L | 0.915962811196283 | 0.6059016814709497 |
### Chart: Cyclin D1_Actin
| Category | HEK293 | M059K |
|---|---|---|
| GFP | 0.2692668247253312 | 0.8484846751548232 |
| MYT1L | 0.34056544388861054 | 0.4595128601881031 |
### Chart: CDK6_Actin
| Category | HEK293 | M059K |
|---|---|---|
| GFP | 1.5370124208001599 | 0.6436424540582374 |
| MYT1L | 1.4320073524693646 | 0.5558263768941629 |**
**
**
*
**
### Chart: Cyclin E1_Actin
| Category | HEK293 | M059K |
|---|---|---|
| GFP | 2.3803089164231133 | 0.5272224416917216 |
| MYT1L | 1.983658683580092 | 0.8776686950574334 |*
**
Supplementary Figure S1

## Slide 4
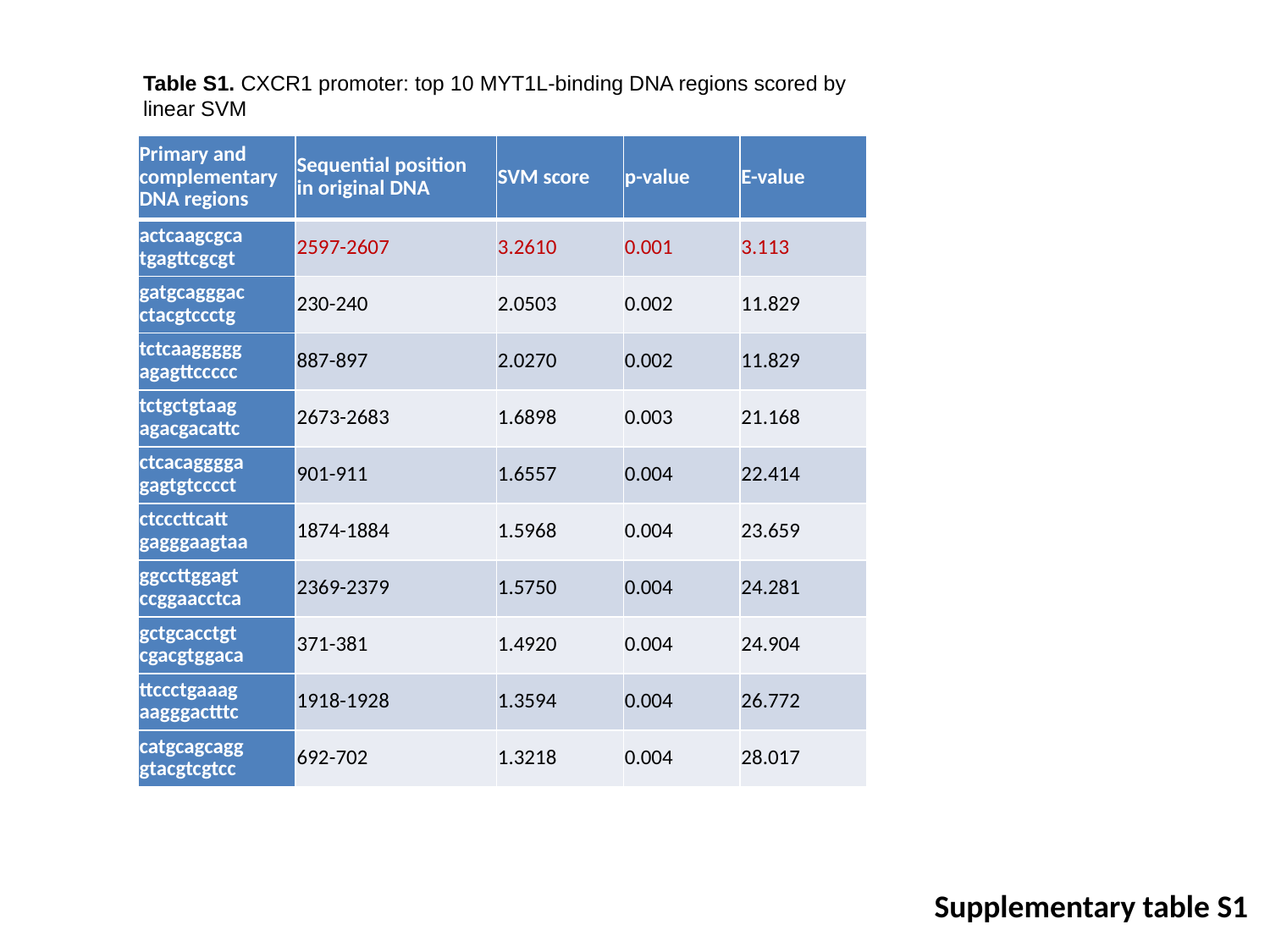

Table S1. CXCR1 promoter: top 10 MYT1L-binding DNA regions scored by
linear SVM
| Primary and complementary DNA regions | Sequential position in original DNA | SVM score | p-value | E-value |
| --- | --- | --- | --- | --- |
| actcaagcgca tgagttcgcgt | 2597-2607 | 3.2610 | 0.001 | 3.113 |
| gatgcagggac ctacgtccctg | 230-240 | 2.0503 | 0.002 | 11.829 |
| tctcaaggggg agagttccccc | 887-897 | 2.0270 | 0.002 | 11.829 |
| tctgctgtaag agacgacattc | 2673-2683 | 1.6898 | 0.003 | 21.168 |
| ctcacagggga gagtgtcccct | 901-911 | 1.6557 | 0.004 | 22.414 |
| ctcccttcatt gagggaagtaa | 1874-1884 | 1.5968 | 0.004 | 23.659 |
| ggccttggagt ccggaacctca | 2369-2379 | 1.5750 | 0.004 | 24.281 |
| gctgcacctgt cgacgtggaca | 371-381 | 1.4920 | 0.004 | 24.904 |
| ttccctgaaag aagggactttc | 1918-1928 | 1.3594 | 0.004 | 26.772 |
| catgcagcagg gtacgtcgtcc | 692-702 | 1.3218 | 0.004 | 28.017 |
Supplementary table S1

## Slide 5
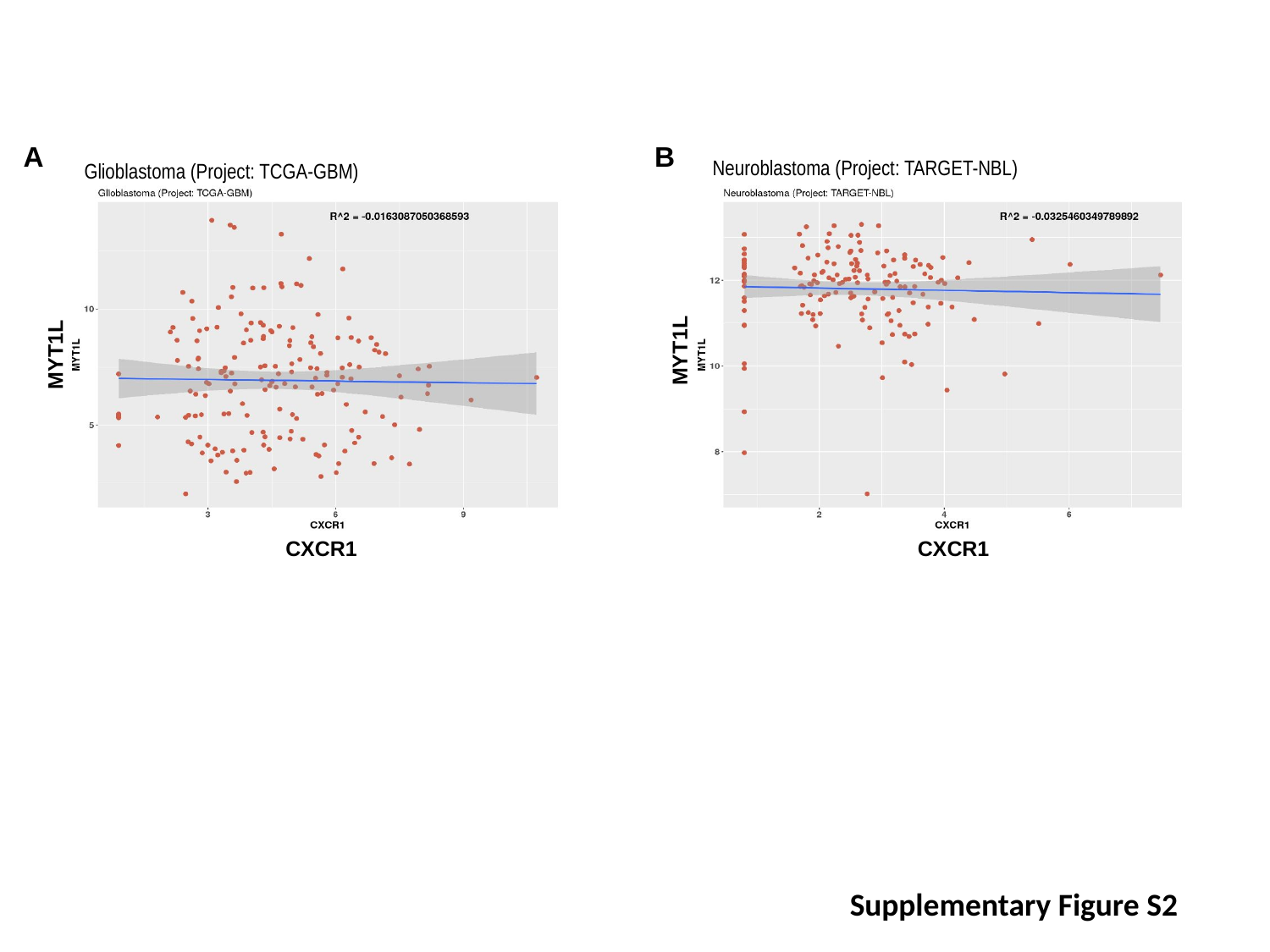

A
B
Neuroblastoma (Project: TARGET-NBL)
Glioblastoma (Project: TCGA-GBM)
MYT1L
MYT1L
CXCR1
CXCR1
Supplementary Figure S2

## Slide 6
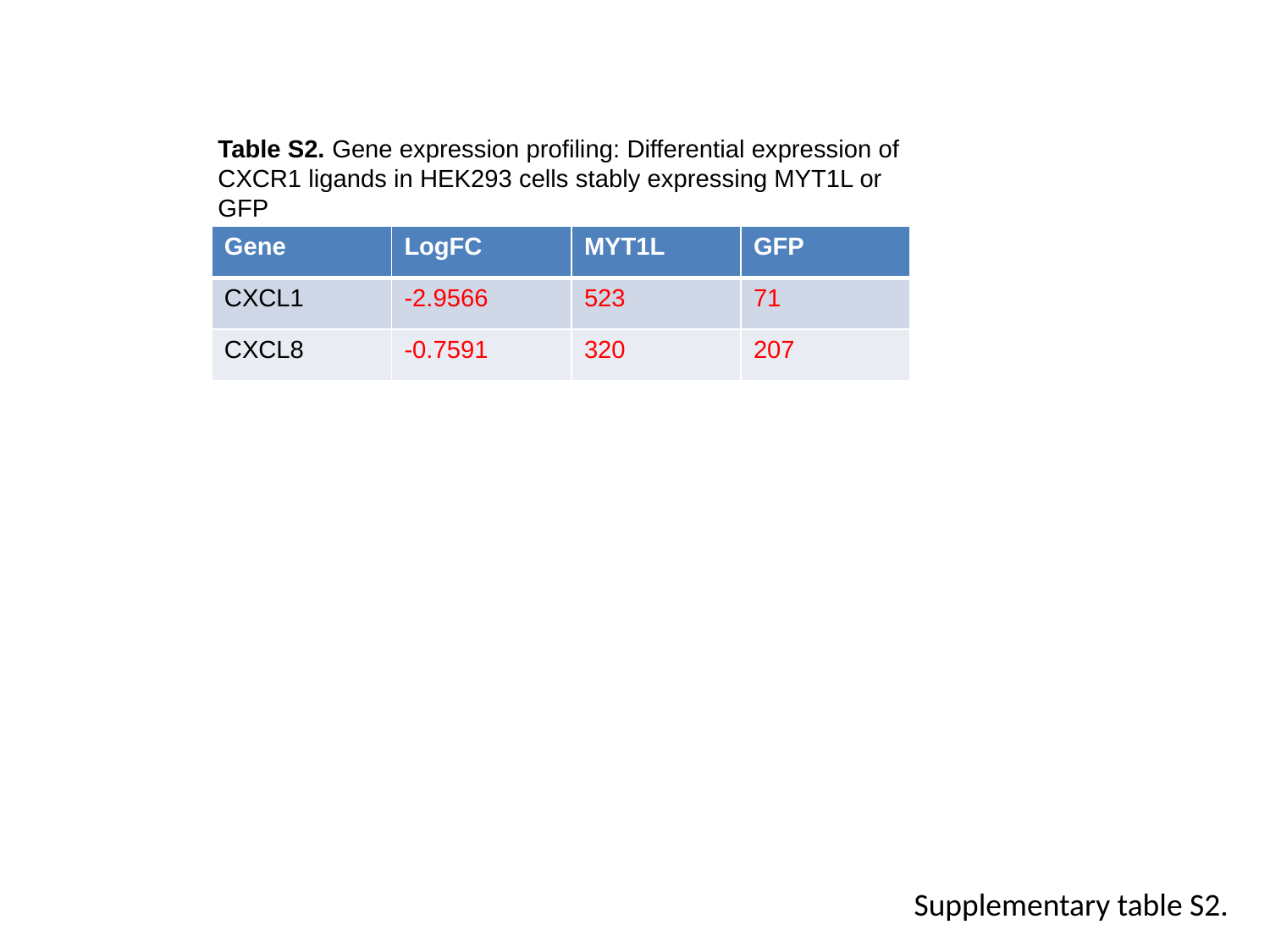

Table S2. Gene expression profiling: Differential expression of CXCR1 ligands in HEK293 cells stably expressing MYT1L or GFP
| Gene | LogFC | MYT1L | GFP |
| --- | --- | --- | --- |
| CXCL1 | -2.9566 | 523 | 71 |
| CXCL8 | -0.7591 | 320 | 207 |
Supplementary table S2.

## Slide 7
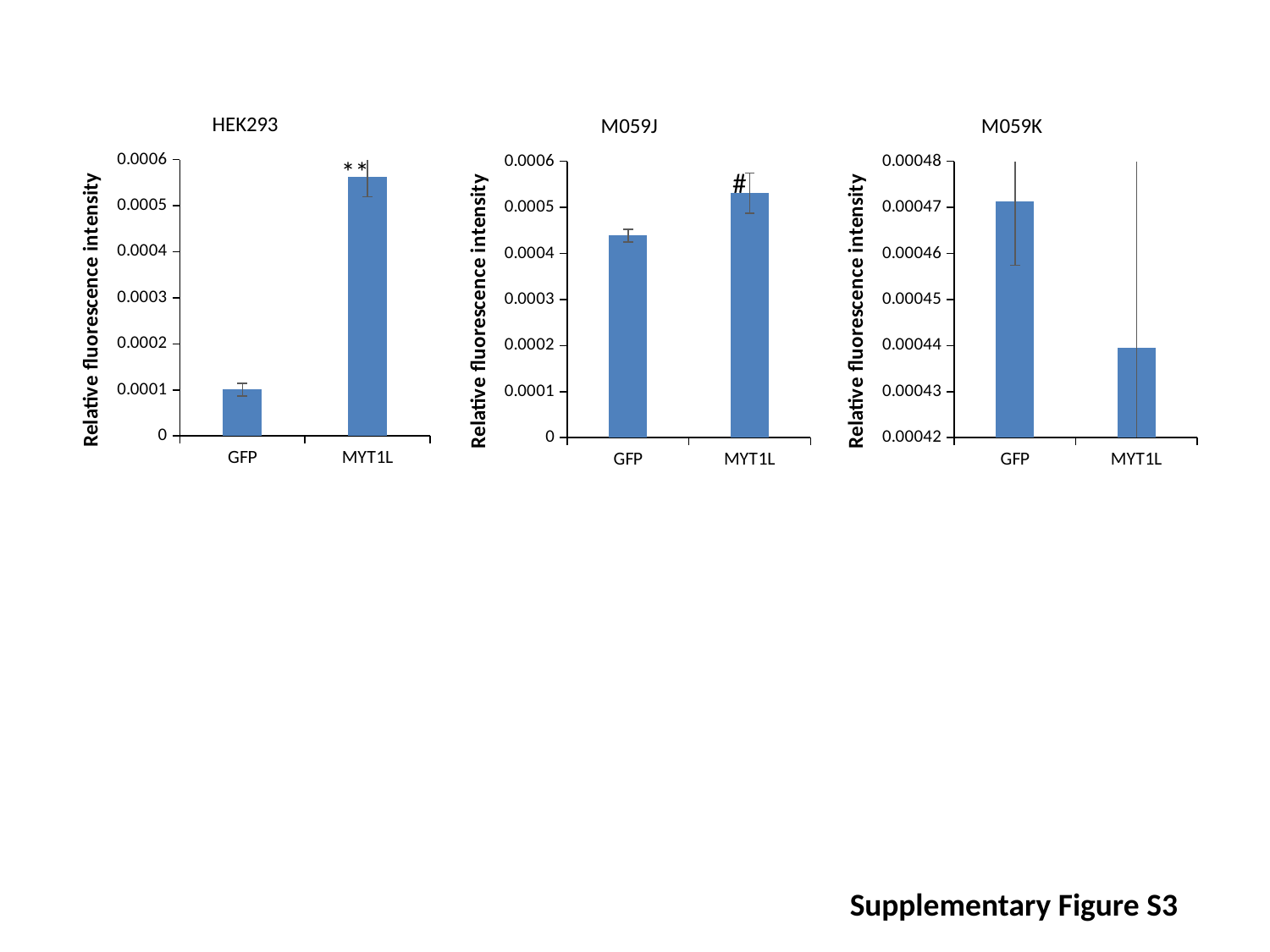

### Chart: HEK293
| Category | |
|---|---|
| GFP | 0.00010077996538624355 |
| MYT1L | 0.0005631130863522699 |
### Chart: M059J
| Category | |
|---|---|
| GFP | 0.00043887712876953866 |
| MYT1L | 0.0005309134168261209 |
### Chart: M059K
| Category | |
|---|---|
| GFP | 0.00047136256242323116 |
| MYT1L | 0.000439514711488666 |**
#
Supplementary Figure S3

## Slide 8
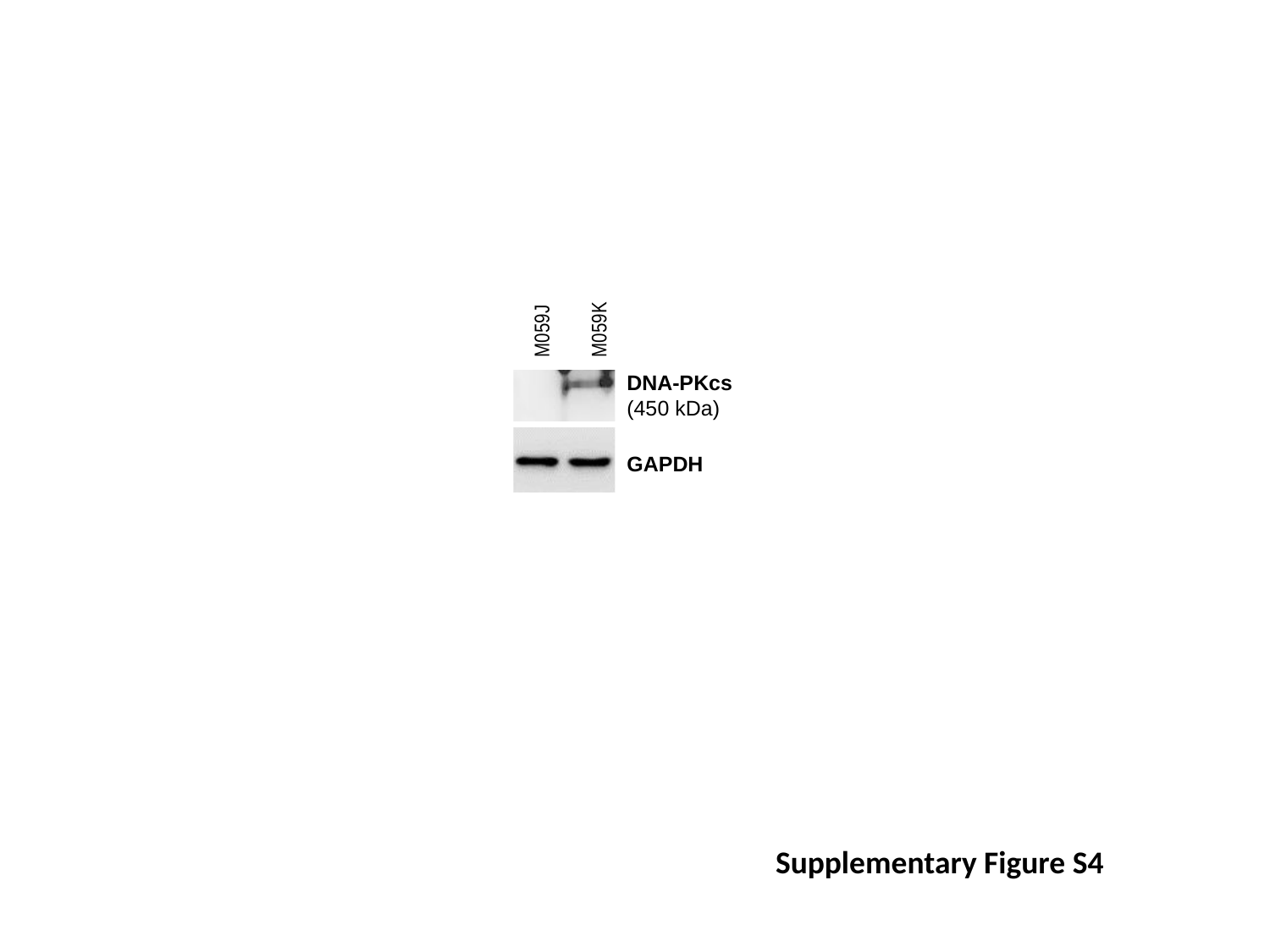

M059J
M059K
DNA-PKcs
(450 kDa)
GAPDH
Supplementary Figure S4

## Slide 9
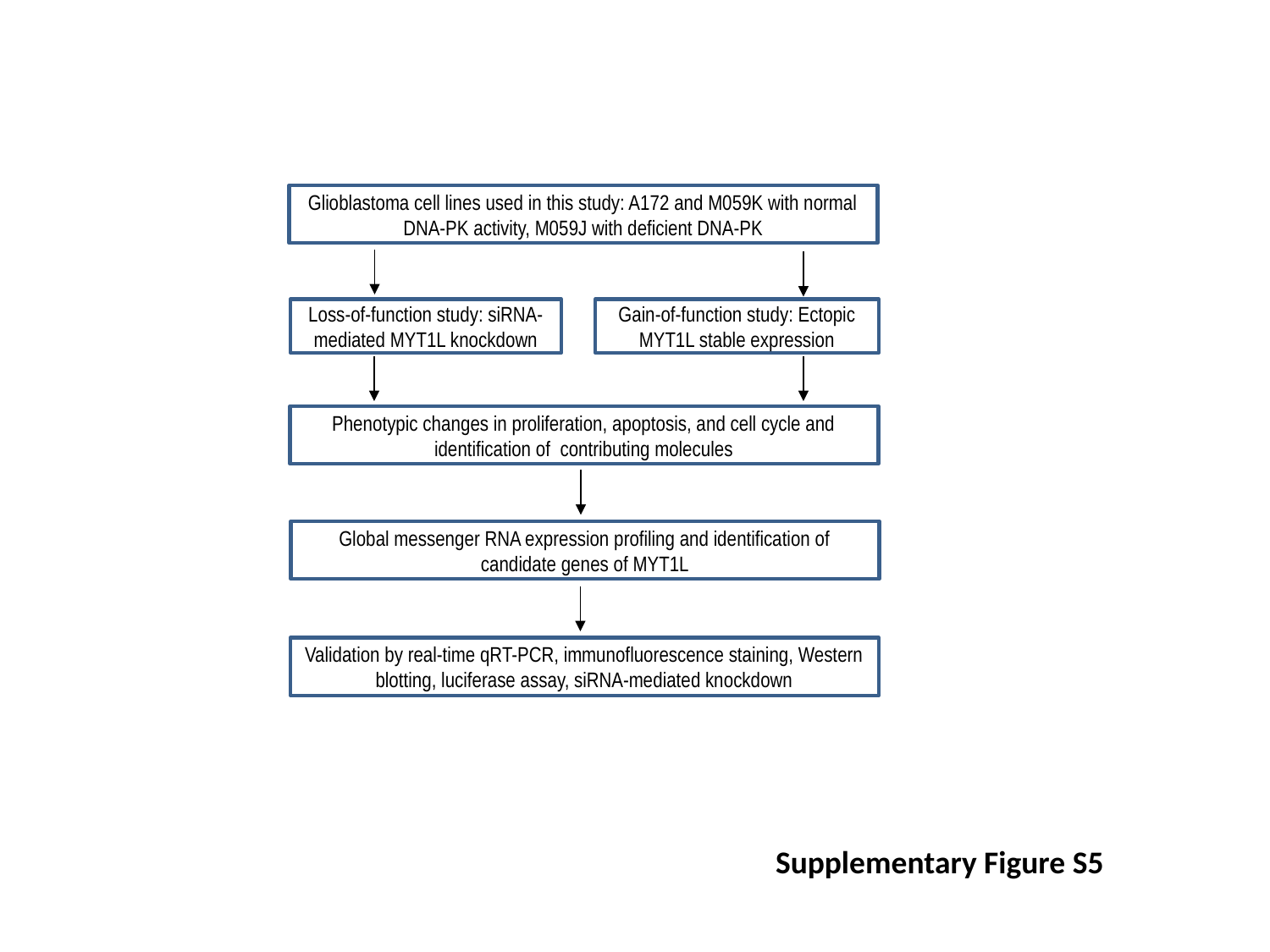

Glioblastoma cell lines used in this study: A172 and M059K with normal DNA-PK activity, M059J with deficient DNA-PK
Loss-of-function study: siRNA-mediated MYT1L knockdown
Gain-of-function study: Ectopic MYT1L stable expression
Phenotypic changes in proliferation, apoptosis, and cell cycle and identification of contributing molecules
Global messenger RNA expression profiling and identification of candidate genes of MYT1L
Validation by real-time qRT-PCR, immunofluorescence staining, Western blotting, luciferase assay, siRNA-mediated knockdown
Supplementary Figure S5
